# Rumen-host crosstalk: an amplifier of systemic pathology in heat-stressed ruminants

**DOI:** 10.3389/fmicb.2026.1912454

**Published:** 2026-08-21

**Authors:** Yuyan Wang, Chunjia Jin, Qian Li, Ye Yu, Binlong Fu, Runqi Fu, Zhengyuan Cai, Xitong An, Jing Leng

**Affiliations:** Yunnan Provincial Key Laboratory of Animal Nutrition and Feed, Faculty of Animal Science and Technology, Yunnan Agricultural University, Kunming, China

**Keywords:** heat stress, neuro-endocrine-immune network, oxidative stress, rumen microbiota, rumen-host crosstalk, ruminants

## Abstract

Heat stress (HS) is a primary environmental constraint threatening ruminant production, welfare and reproductive efficiency amid global warming. Current studies mostly focus on either systemic physiological damage or local ruminal dysfunction separately, while the bidirectional crosstalk between the rumen and host, as well as its amplifying effect on systemic pathology, remains to be systematically elucidated. This review integrates the latest evidence to construct a mechanistic framework centered on the neuro-endocrine-immune network, which acts as the core hub mediating rumen-host interactions under HS. Upon HS exposure, hyperactivation of the hypothalamic–pituitary–adrenal (HPA) and sympatho-adreno-medullary (SAM) axis drives systemic oxidative stress, immune dysfunction and chronic low-grade inflammation, disrupting whole-body homeostasis. Concurrently, HS reshapes the structure and functional profiles of rumen microbiota via three synergistic pathways: direct thermal sensing, host neuroendocrine regulation, and ruminal microenvironmental disturbance, thereby further impairing rumen fermentation homeostasis and epithelial barrier integrity, and triggering pathological endotoxin translocation. Critically, the neuro-endocrine-immune network transduces localized ruminal pathological signals into systemic metabolic and inflammatory disorders, and forms a self-perpetuating vicious cycle: systemic stress in turn aggravates ruminal ischemia, microbial dysbiosis and barrier damage, amplifying the initial pathological damage. By delineating this rumen-host pathological amplifier framework, this review provides targeted theoretical guidance for the development of multi-node intervention strategies to mitigate HS-associated production losses in ruminants.

## 1 Introduction

Global climate change is accelerating rapidly, and rising ambient temperatures coupled with high humidity act as critical environmental stressors that negatively impact ruminant farming systems. Such thermal stressors severely compromise livestock welfare, productivity and reproductive performance (Shabtay, 2025). Heat stress (HS) is a non-specific physiological reaction to thermal dysregulation. It disrupts the homeostatic mechanisms vital to ruminant health, rendering these animals particularly vulnerable to metabolic collapse during summer production periods (Silva Neto et al., 2025). The pathophysiological cascade triggered by heat stress is directly associated with impaired thermoregulation, heightened oxidative stress and inflammatory responses, as well as reduced feed intake, altered nutrient allocation, and growth suppression. This simultaneously compromises both economic efficiency and animal welfare (Yaghoobpour et al., 2025). With ongoing global warming, heat stress has emerged as a primary threat to livestock production worldwide (Bernabucci et al., 2010; Fan and McColl, 2024).

Ruminants, particularly heat-sensitive species such as dairy cows and sheep, exhibit heightened susceptibility to thermal challenges due to their unique physiological architecture (Gonzalez-Rivas et al., 2020). Ruminal fermentation processes are known to yield high basal metabolic rates (Deng et al., 2018). Compounding this issue is the limited efficacy of evaporative cooling in ruminants, a constraint attributed to their dense pelage (Berman, 2009). A further contributing factor is the substantial heat these animals generate, heat exceeding their capacity for dissipation via radiation, convection, or conduction (Hassan et al., 2025). This thermoregulatory imbalance precipitates core body temperature elevations exceeding physiological thresholds, triggering compensatory mechanisms that divert energy reserves from productive functions to heat mitigation strategies (Abdel-Hamied et al., 2025).

The impact of heat stress on the organism is systemic, encompassing the activation of neuroendocrine stress responses, induction of oxidative damage, weakening of immune defenses, disruption of intestinal barrier integrity, and perturbation of metabolic homeostasis, among other aspects (Gonzalez-Rivas et al., 2020). The rumen is recognized as the central component of the ruminant digestive system and undergoes changes to its intricate microbial ecology when exposed to heat stress. These ecological shifts, in turn, exert an influence on host physiological status through the production of metabolites and inflammatory mediators (Chen et al., 2026). The host’s neuroendocrine stress responses shape the rumen microbial community, with microbes and their products modulating the host’s immune and metabolic reactions (Jiang X. et al., 2026). Animals under such conditions show reduced rumination efficiency, compromised immune competence, and altered microbiota dynamics. These changes lead to metabolic perturbations, forming a vicious cycle that increases the animals’ disease susceptibility.

**Figure 1:**
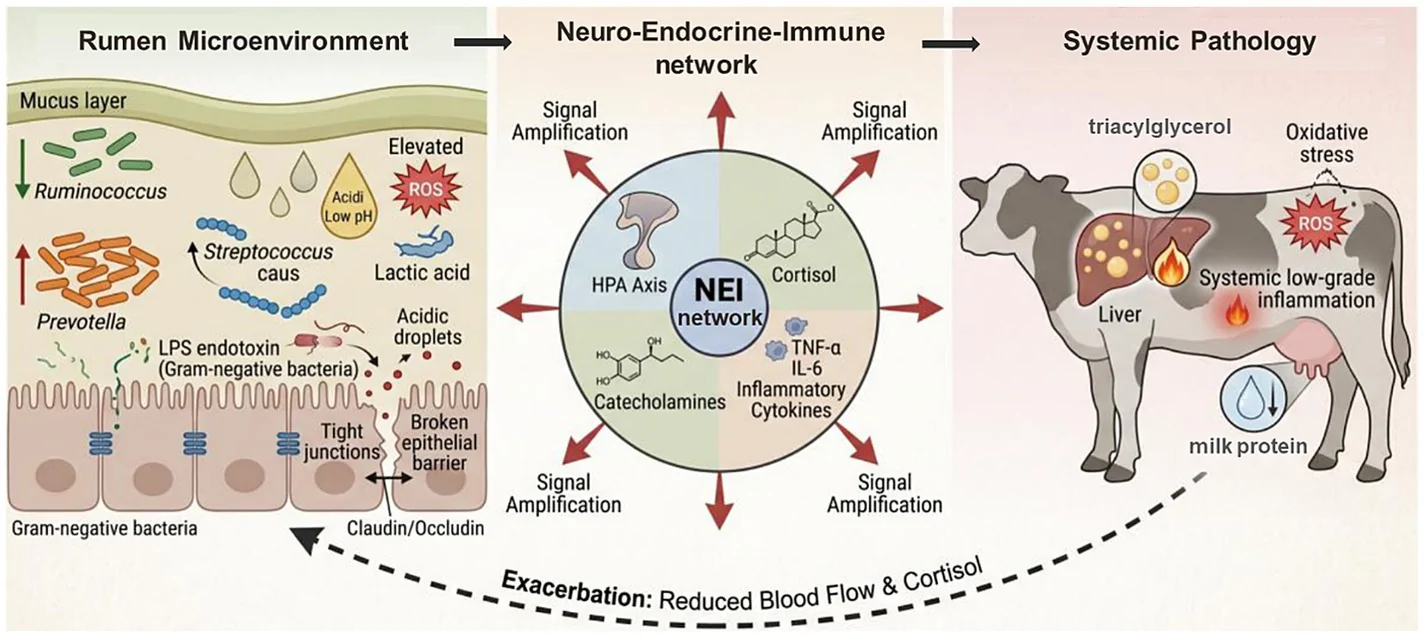
Heat stress mechanisms in ruminants: Rumen microbial ecosystem and host interactions.

This review sets out to systematically elaborate on the mechanisms that drive key physiological alterations in ruminants during heat stress. It also addresses the multifaceted crosstalk between these physiological responses and the rumen microbial ecosystem. The overall bidirectional pathological interaction between rumen microenvironment and systemic host pathways is illustrated in Figure 1, which visually frames the rumen-host crosstalk as an integrated, bidirectional circuit rather than separate local and systemic events. The schematic highlights that microbial dysbiosis and epithelial barrier disruption are not confined to the rumen but actively propagate pathological signals to the host via humoral and neural routes. This integrated perspective sets the stage for understanding how the neuro-endocrine-immune network serves as the central hub that coordinates this bidirectional communication.

## 2 The main physiological pathways induced by heat stress and their impact on homeostasis

### 2.1 Neuroendocrine regulation and systemic thermoregulatory response

HS describes a nonspecific systemic response that sets in when external thermal stimuli outstrip the body’s heat dissipation capacity, breaking the balance between endogenous heat production and heat loss (Muhamad et al., 2025). In ruminants, heat stress occurs most frequently under hot, humid conditions, and its trigger threshold varies widely by species, breed, age and production stage (Gupta et al., 2025). High-yielding dairy breeds, especially Holstein cows, show the highest sensitivity to heat stress. Their small surface area per unit body weight, dense hair coat and underdeveloped sweat glands collectively limit evaporative heat loss, making endogenous heat buildup particularly likely (Bernabucci et al., 2010). The neuroendocrine system acts as the core regulatory axis for the whole-body heat stress response, working mainly through coordinated activation of the hypothalamic–pituitary–adrenal (HPA) axis and the sympatho-adreno-medullary (SAM) system.

#### 2.1.1 HPA axis activation and cortisol-mediated physiological effects

When the hypothalamic thermoregulatory center detects a rise in core body temperature, it activates corticotropin-releasing hormone (CRH) neurons in the paraventricular nucleus, which in turn drives the anterior pituitary to secrete adrenocorticotropic hormone (ACTH). ACTH then stimulates the adrenal cortex to synthesize and secrete glucocorticoids, with cortisol as the core effector molecule (Horváth et al., 2023).

Cortisol directly suppresses neuropeptide Y (NPY) expression in the hypothalamic arcuate nucleus, while boosting anorexigenic signaling from pro-opiomelanocortin (POMC), together driving a marked drop in dry matter intake (DMI) (Mbiydzenyuy and Qulu, 2024). Meta-analysis data show that for every 1-unit rise in temperature-humidity index (THI) in mid-lactation cows, DMI and energy-corrected milk (ECM) yield fall by 4.13 and 3.25%, respectively (Chen et al., 2024). Cortisol promotes skeletal muscle protein breakdown and adipose tissue lipolysis, mobilizing endogenous nutrient reserves and inducing peripheral insulin resistance to prioritize glucose delivery to vital organs like the brain and heart. This adaptive response triggers systemic metabolic disorders, including elevated serum non-esterified fatty acids, hepatic triacylglycerol buildup, and reduced milk protein synthesis rates (Min et al., 2017).

In dairy cows, sustained neuroendocrine dysregulation typically causes a 10–20% drop in milk yield during heat stress (Amato et al., 2025). Excess cortisol inhibits the hypothalamic–pituitary-gonadal axis, downregulates luteinizing hormone (LH) receptor expression in ovarian granulosa cells, and upregulates prostaglandin F2α (PGF2α) production. This suppresses luteal function and cuts progesterone synthesis, ultimately impairing embryo implantation and early pregnancy maintenance (Batool et al., 2025). Chronic heat stress also disrupts placental nutrient transport by downregulating insulin-like growth factor-1 (IGF-1) signaling and amino acid transporters solute carrier family 7 member A7 (SLC7A7) and glucose transporter 12 (GLUT12), hindering fetal development (Capela et al., 2025; Casarotto et al., 2025).

#### 2.1.2 SAM system activation and catecholamine-mediated effects

HS also activates the SAM, triggering the release of epinephrine and norepinephrine from chromaffin cells in the adrenal medulla. These catecholamines bind to adrenergic receptors in target tissues to exert rapid regulatory effects (Motiejunaite et al., 2021).

Catecholamines bind to beta-1 adrenergic receptors (*β*₁-AR) in cardiac tissue, raising heart rate and strengthening myocardial contractility to boost cardiac output. High humidity cuts down sweat evaporation efficiency, so animals compensate with hyperventilation to dissipate heat through latent heat loss from the respiratory mucosa. This response can lead to compensatory respiratory alkalosis (Bright et al., 2025).

Catecholamines stimulate lipolysis and hepatic gluconeogenesis via the cyclic adenosine monophosphate (cAMP)-dependent protein kinase A (PKA) signaling pathway, and suppress insulin secretion to maintain blood glucose homeostasis. Simultaneously, they activate mitochondrial nicotinamide adenine dinucleotide phosphate (NADPH) oxidase, leading to reactive oxygen species (ROS) production, which serves as a key trigger of systemic oxidative stress (Reilly et al., 2020). Catecholamines induce vasoconstriction in the gastrointestinal tract, cutting rumen epithelial blood flow by 30–40% (Huang et al., 2018). This adaptive response ensures blood supply to vital organs, but directly disrupts the ruminal anaerobic microenvironment and impairs epithelial barrier integrity, which serves as a key link connecting systemic stress to rumen dysfunction.

Taken together, the neuroendocrine system coordinates thermoregulation and metabolic adaptation on one hand, and drives immunosuppression, metabolic disorder and rumen damage on the other. Sustained overactivation of the stress axis pushes the body from physiological adaptation to pathological damage.

### 2.2 Oxidative stress, cellular heat shock response and tissue damage

Prolonged heat stress drives excess free radical production and imbalances the endogenous antioxidant defense system, together triggering oxidative stress, which serves as a core pathological mechanism that mediates cell damage, tissue dysfunction and systemic inflammatory response (Forman and Zhang, 2021). In parallel, cells activate a conserved heat shock response as an adaptive defense against thermal and oxidative damage.

#### 2.2.1 Oxidative stress induction and antioxidant system imbalance

Under HS, multiple pathways including mitochondrial respiratory chain dysfunction, catecholamine oxidation and inflammatory cell activation jointly promote the excessive production of ROS such as superoxide anions, hydrogen peroxide and hydroxyl free radicals.

When the generation rate of ROS exceeds the scavenging capacity of the endogenous antioxidant system, oxidative stress occurs (Jiang Q. et al., 2026). The enzymatic antioxidant system, including superoxide dismutase (SOD), glutathione peroxidase (GSH-Px) and catalase (CAT), is significantly inhibited under heat stress, and the level of non-enzymatic antioxidants such as glutathione is also depleted. Malondialdehyde (MDA), a product of lipid peroxidation, accumulates significantly in serum and tissues, which is a classic biomarker of oxidative damage (Li et al., 2025d). In ruminants, heat stress-induced oxidative stress shows systemic distribution characteristics. Studies on dairy cows have detected consistent changes in antioxidant indexes and oxidative damage products in both serum and follicular fluid, accompanied by elevated levels of pro-inflammatory cytokines, including interleukins (ILs) such as IL-1, IL-6, and tumor necrosis factor-alpha (TNF-*α*), as well as heat shock protein 70 (HSP70) and heat shock protein 90 (HSP90), indicating that oxidative stress is widely involved in the pathological process of multiple tissues (Matas-Quintanilla et al., 2026; Yan et al., 2026).

#### 2.2.2 Cellular heat shock response and adaptive protection

To cope with thermal and oxidative damage, cells initiate a conserved heat shock response, which is centered on the synthesis of heat shock proteins (HSPs) and maintains cellular proteostasis (Hu et al., 2022).

Under physiological conditions, heat shock factor 1 (HSF1) exists as an inactive monomer in the cytoplasm by binding to HSP70 and HSP90. Heat stress induces protein denaturation and aggregation, and exposed hydrophobic domains compete with HSF1 for binding to molecular chaperones, leading to HSF1 release and trimerization. Trimeric HSF1 translocates to the nucleus, undergoes phase separation to form nuclear condensates, and binds to heat shock elements (HSEs) in the promoter region of HSP genes. Post-translational modifications (Ser230 phosphorylation and C-terminal acetylation) further enhance HSF1 activity, driving robust transcription of HSP genes (Ahn and Im, 2020; Pessa et al., 2024). When stress is relieved, accumulated HSP70 and HSP90 bind to HSF1 again to promote its depolymerization and nuclear export, forming a negative feedback loop to terminate excessive transcription (Kijima et al., 2018).

As molecular chaperones, HSPs exert cytoprotective effects through multiple pathways. They assist the folding of nascent polypeptides to prevent protein aggregation and mediate the degradation of irreparably damaged proteins to recycle amino acids. Beyond maintaining cellular proteostasis, they stabilize mitochondrial membrane structure to reduce ROS leakage and activate the phosphatidylinositol 3-kinase (PI3K)/protein kinase B (Akt) signaling cascade to inhibit caspase-3 cleavage and exert anti-apoptotic effects (Chatterjee et al., 2013; Wan et al., 2020; Singh et al., 2025). The heat tolerance phenomenon, in which sublethal pre-exposure improves survival under lethal heat shock, is mainly mediated by HSP70-induced protein repair and cellular structure stabilization (Li et al., 2018).

Notably, HSP expression shows spatiotemporal specificity in rumen epithelial cells. HSP70 expression peaks at 24 h post heat stress at levels two to three fold above baseline, a phenomenon induced by co-activation of nuclear factor kappa-B (NF-κB) and HSF1 (Muhamad et al., 2025). This adaptive response can stabilize denatured proteins and inhibit apoptosis mediated by caspase-3 (CASP3) and caspase-8 (CASP8), which is an important defense mechanism for rumen epithelium to resist heat stress damage (Singh et al., 2024).

#### 2.2.3 Oxidative stress-mediated tissue damage

When oxidative stress exceeds the cellular adaptive defense capacity, it will cause irreversible damage to multiple tissues, among which the reproductive system is highly sensitive to oxidative damage.

In females, excessive ROS induces oxidative damage to ovarian follicles, disrupts follicular development and oocyte maturation, and impairs preimplantation embryo development (Lei et al., 2026; Ramírez et al., 2026). At the molecular level, heat stress inhibits the expression of cell differentiation-related genes, including Wnt family member 5A (WNT5A) and notch receptor 1 (NOTCH1), in granulosa cells. Although activated HSP70/HSP90 chaperone systems alleviate proteotoxicity, they simultaneously disrupt the expression of steroidogenic enzymes such as cytochrome P450 family 11 subfamily A member 1 (CYP11A1) and steroidogenic acute regulatory protein (STAR), leading to decreased progesterone biosynthesis (Yu X. et al., 2025). In males, testicular hyperthermia induced by HS impairs spermatogenesis: it induces Sertoli cell apoptosis and Leydig cell dysfunction, reduces sperm motility and seminal plasma antioxidant capacity, and causes sperm DNA fragmentation (Wang et al., 2024; Alves et al., 2025). Non-targeted metabolomics studies have shown that heat stress induces systemic remodeling of the arachidonic acid metabolic pathway, and that ferroptosis-related genes are abnormally expressed, with acyl-CoA synthetase long-chain family member 4 (ACSL4), transferrin receptor 1 (TfR1), cyclooxygenase-2 (COX2), and ChaC glutathione-specific gamma-glutamylcyclotransferase 1 (CHAC1) being upregulated, whereas glutathione peroxidase 4 (GPX4), solute carrier family 7 member 11 (SLC7A11), and regulator of G protein signaling 4 (RGS4) are downregulated, indicating that oxidative stress drives cell death through multiple programmed pathways (Zhou et al., 2026).

From the perspective of the whole pathological cascade, oxidative stress is not only a consequence of heat stress-induced metabolic disorder, but also a key mediator connecting cellular damage to systemic immune inflammation and metabolic dysfunction. Sustained oxidative stress will further aggravate tissue damage and reduce production performance, which is an important target for nutritional intervention to alleviate heat stress (Zhang et al., 2025).

### 2.3 Immune dysfunction and inflammatory response

HS impacts the ruminant immune system in complex ways, who’s main feature is immunosuppression accompanied by mild systemic inflammation (Cantet et al., 2021). It activates the HPA axis, prompting excessive glucocorticoid release. This release exerts widespread immunosuppressive effects (Douglass et al., 2023). Specifically, cortisol inhibits immune cell proliferation and antigen presentation, weakens the functional activity of T lymphocytes and macrophages, and disrupts the synthesis and secretion of various cytokines (Taves and Ashwell, 2021). Previous studies have confirmed that the production of pro-inflammatory and antiviral cytokines, such as TNF-*α*, IL-4, IL-12, and interferon-gamma (IFN-*γ*), in peripheral blood is significantly inhibited by elevated cortisol levels in heat-stressed dairy cows (Choudhary et al., 2026).

HS induces a functional shift in T helper cell subsets. Specifically, it suppresses T helper 1 (Th1)-mediated cellular immunity while relatively enhancing T helper 2 (Th2)-mediated humoral immunity, leading to an imbalanced Th1/Th2 cytokine profile (Heintzman et al., 2024). Th2-related cytokines such as IL-10 and IL-4 are upregulated, whereas the expression of Th1-related cytokines including IFN-γ and IL-12 is downregulated. This shift suppresses cellular immune responses, while boosting humoral immunity and anti-inflammatory reactions (Gocher-Demske et al., 2023). Such immune redistribution results in compromised cellular immunity and reduced anti-infective capacity, increasing susceptibility to diseases. In heat-stressed dairy cows, leukocyte function is impaired, phagocytic activity is reduced, and lymphocyte proliferation is weakened, with research confirming these changes are closely linked to glucocorticoid-mediated immunosuppression (Yu Z. et al., 2025). HS induces systemic immunosuppression in the animals. This impairs host resistance to pathogens and increases the likelihood of infectious diseases.

Heat stress often leads to low-grade systemic inflammation. This inflammation is mainly caused by tissue damage and increased intestinal permeability, which permit endotoxins and other inflammatory mediators to enter the circulation and trigger inflammatory cascades (Abdelmegeid et al., 2026). Growing evidence shows that heat stress raises blood cortisol levels. These elevated cortisol levels can suppress the synthesis of various cytokines, including IL-4, IL-5, IL-6, IL-12, IFNγ, and TNF-*α* (Bagath et al., 2019). This phenomenon can be partially explained by oxidative stress-induced tissue damage and the release of inflammatory mediators, and is also closely associated with “leaky gut” syndrome and endotoxemia caused by impaired intestinal barrier function (Schroeder et al., 2024).

Transcriptome sequencing confirms that heat stress boosts the expression of inflammation-related genes in peripheral leukocytes. Bradykinin receptor B1 (B1R) is one such gene, and this upregulation points to accelerated inflammatory signaling caused by tissue injury and increased vascular permeability (Garner et al., 2020). Pro-inflammatory mediators increase under heat stress, yet the potent immunosuppressive effect of cortisol markedly impairs the host’s capacity to respond to and clear these inflammatory signals (Megha et al., 2021). This leads to “chronic systemic low-grade inflammation” a state with high inflammatory markers but poor immune clearance. The coexistence of immunosuppression and low-grade inflammation makes heat-stressed animals prone to subclinical inflammation (Wu et al., 2026). They also struggle to eliminate pathogenic infections, which in turn impairs their health and productivity.

**Figure 2:**
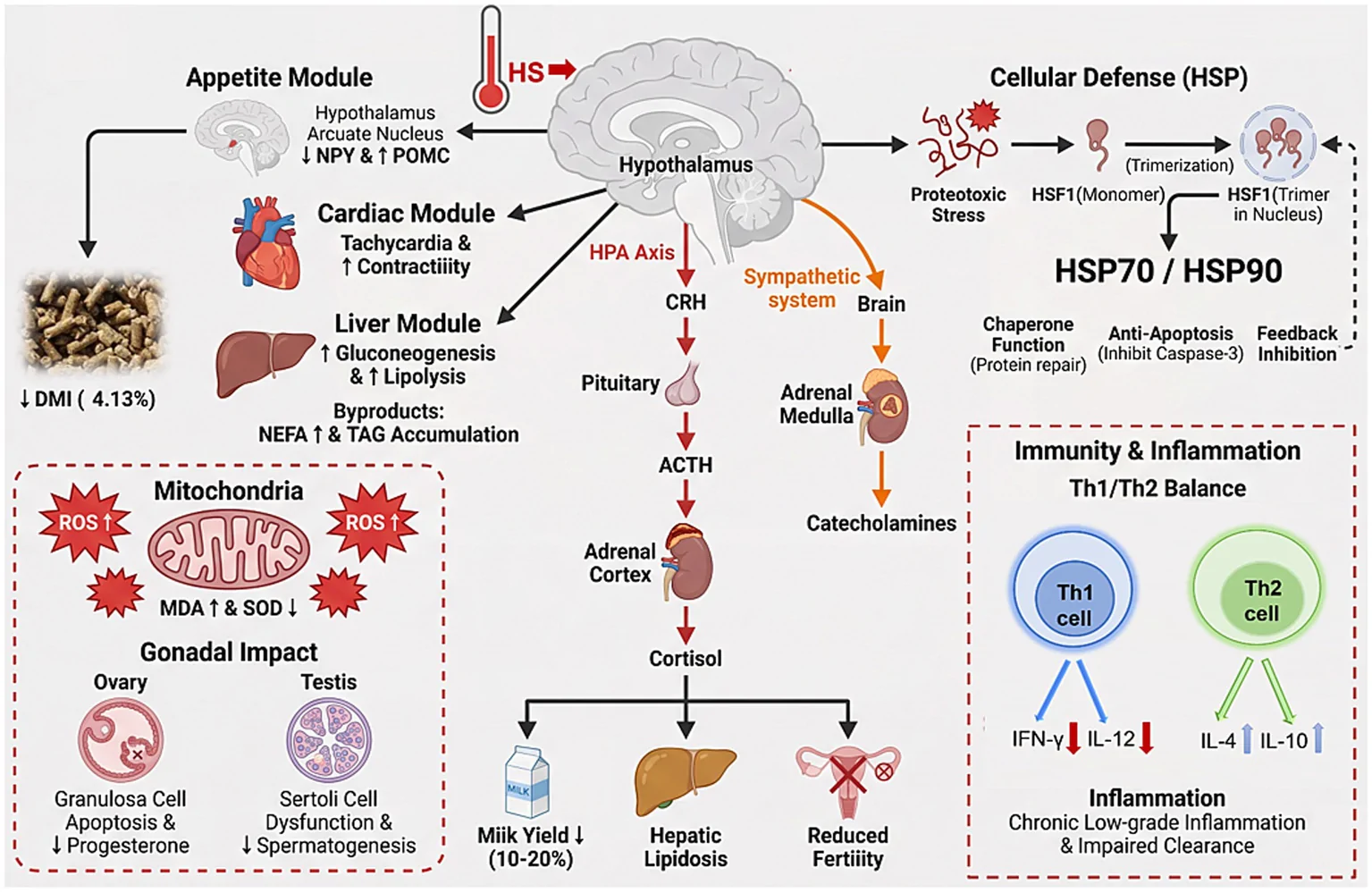
Mechanisms of heat stress-induced homeostatic imbalance in ruminants.

The key molecular alterations and corresponding biological effects of the major physiological pathways in heat-stressed ruminants are systematically summarized in Table 1. Thus, heat stress mediates immunosuppression via neuroendocrine pathways and triggers inflammation through oxidative injury and barrier dysfunction, collectively disrupting immune homeostasis and defensive function in ruminants. The integrated cascade of neuroendocrine activation, oxidative damage, heat shock response and immune imbalance induced by heat stress is comprehensively summarized in Figure 2, which maps the temporal progression from initiating stress signals to downstream homeostatic failure. The figure clarifies that hyperactivation of the HPA and SAM axes acts as the primary trigger and sequentially induces oxidative stress, heat shock responses, and immunosuppression. The schematic displays the hierarchical structure of this cascade and demonstrates that early adaptive reactions such as elevated HSP expression are gradually suppressed by persistent pathological stimuli, eventually resulting in systemic homeostatic collapse.

**Table 1:** Main physiological alterations in heat-stressed ruminants.

Regulatory pathway category	Key molecules	Change under heat stress	Biological effects
Neuroendocrine regulatory axis	Cortisol, ACTH, CRH	Significantly up-regulated	Reduced feed intake; metabolic reprogramming; immunosuppression; splanchnic vasoconstriction; reproductive impairment (Huang et al., 2018; Chen et al., 2024; Batool et al., 2025)
Neuroendocrine regulatory axis	Epinephrine, Norepinephrine	Up-regulated	Tachycardia; respiratory alkalosis; stimulated lipolysis & gluconeogenesis; ROS overproduction; gastrointestinal vasoconstriction (Huang et al., 2018; Reilly et al., 2020; Bright et al., 2025)
Oxidative stress response	SOD, GSH-Px, CAT; MDA	Antioxidant enzymes down-regulated; MDA accumulated	Systemic oxidative damage; lipid peroxidation; cell apoptosis; tissue injury (Li et al., 2025d; Jiang Q. et al., 2026)
Oxidative stress response	HSP70, HSP90, HSF1	Significantly up-regulated (adaptive response)	Maintained proteostasis; anti-apoptosis; improved heat tolerance; rumen epithelium protection (Hu et al., 2022; Singh et al., 2024)
Immune function alteration	TNF-*α*, IL-1β, IL-6	Up-regulated (chronic low-grade inflammation)	Systemic low-grade inflammation; metabolic disorder; aggravated barrier damage (Bagath et al., 2019; Cantet et al., 2021)
Immune function alteration	Th1 cytokines (IFN-γ, IL-12); Th2 cytokines (IL-4, IL-10)	Th1 response suppressed; Th2 response relatively enhanced	Impaired cellular immunity; reduced anti-infective capacity; increased disease susceptibility (Heintzman et al., 2024; Yu Z. et al., 2025)

## 3 The impact of heat stress on the rumen microecosystem

The rumen microecosystem is the core site for nutrient digestion and energy acquisition in ruminants. Heat stress disrupts rumen homeostasis through multi-level effects. It alters microbial sensing and regulatory pathways, reshapes community structure, and impairs fermentation and barrier functions. These local changes further drive systemic physiological disorders through bidirectional crosstalk with the host.

### 3.1 Molecular sensing and regulatory mechanisms of rumen microbiota in response to heat stress

Rumen microorganisms perceive and respond to heat stress through three independent but synergistic pathways. These include direct thermal signal sensing, indirect regulation mediated by host neuroendocrine factors, and synergistic sensing of rumen microenvironmental disorders. These mechanisms jointly drive community reorganization and functional remodeling of rumen microbiota, and further regulate host physiological homeostasis through microbe-host interactions (Liu et al., 2021).

#### 3.1.1 Direct thermal signal sensing pathway

In the early stage of heat stress, rumen bacteria directly perceive elevated temperatures through self-encoded heat-sensing elements. RNA thermometers are cis-acting regulatory elements in the 5′-untranslated region of heat shock-related mRNAs. They change shape when heated, exposing the ribosome binding site. This allows quick translation of the heat shock sigma factor rpoH (σ^32^) without new transcription. This is the earliest activated heat-sensing pathway in rumen microbiota (Kortmann and Narberhaus, 2012). Transcriptomic studies have confirmed that RNA temperature sensors are widely present in core rumen bacteria (including Prevotella, Ruminococcus, and Fibrobacter), and that there are significant differences in heat sensitivity among different species, which contributes to microbial differentiation under heat stress conditions (Loh et al., 2018).

The σ^32^-mediated heat shock response is the core regulatory hub for rumen microbes to adapt to thermal stress. Under normal physiological conditions, the DnaK-DnaJ-GrpE chaperone complex functions to sequester σ^32^ and facilitate its degradation. When heat stress is imposed, unfolded proteins compete with σ^32^ for binding to the chaperone complex, thereby releasing free σ^32^ molecules. These free σ^32^ molecules then bind to RNA polymerase, triggering the transcription of molecular chaperones (DnaK, GroEL, HtpG) and proteases to sustain cellular proteostasis (Straus et al., 1987). Metagenomic and metatranscriptomic studies on heat-stressed dairy cows have yielded key findings, including that fibrolytic bacteria only harbor one copy of the rpoH gene with low regulatory redundancy, which prevents them from effectively activating the heat shock response under thermal stress. On the other hand, heat-tolerant taxa like Prevotella have multiple copies of heat shock-related genes. These genes enable robust activation of the σ^32^ pathway, allowing Prevotella to become dominant under heat stress (Miwa and Taguchi, 2023).

In addition, membrane-bound histidine kinases in two-component systems (TCS) sense temperature-induced changes in membrane fluidity, and transduce thermal signals into intracellular transcriptional responses via phosphorelay, which acts as a key bridge connecting temperature sensing and phenotypic adaptation in rumen bacteria (Jacob-Dubuisson et al., 2018).

#### 3.1.2 Indirect regulation mediated by host neuroendocrine pathways

Host neuroendocrine signals can cross the rumen epithelium into the lumen and directly act on microbial cells to regulate their stress response and proliferation. This pathway bridges host systemic stress state and microbial adaptive changes, with HPA axis-cortisol signaling as the core regulatory mediator (Nunez et al., 2025). During heat stress, the host’s adrenal cortex secretes cortisol. This cortisol can cross the rumen epithelium, enter the rumen lumen, and exert direct regulatory effects on rumen microbes. Cortisol enters Gram-negative bacteria via outer membrane porins, directly inhibiting the transcriptional activity of rpoH in fibrolytic bacteria, drastically reducing their heat stress tolerance (Roncarati and Scarlato, 2017). Cortisol activates glucocorticoid-responsive elements in the genomes of Prevotella and other heat-tolerant bacteria, and this direct activation promotes the proliferation of these bacteria (Syed et al., 2020). This process is the core host-driven mechanism for phylum-level microbial differentiation under heat stress.

Meanwhile, neuroendocrine activation triggers splanchnic vasoconstriction and reduces rumen epithelial blood perfusion. This process disrupts the physicochemical state of the rumen internal environment, and these environmental changes are further recognized by microbial sensing systems as indirect heat stress signals, forming a dual regulatory effect of “direct hormone action + indirect environmental remodeling”.

#### 3.1.3 Synergistic sensing of rumen microenvironmental disorders

Heat stress disrupts multiple physicochemical parameters of the rumen internal environment. Rumen microbes sense these environmental disturbances through specific sensing systems, and further adjust their metabolic strategies and survival status accordingly.

Heat stress reduces salivary secretion by more than 40% in ruminants, leading to decreased buffering capacity and lactic acid accumulation, with ruminal pH dropping below 5.5 (Kim et al., 2022). Rumen bacteria sense extracellular acidification via glutamate decarboxylase (Gad) system, arginine deiminase (ADI) system and pH-sensitive TCS, and upregulate acid tolerance-related genes. Fibrolytic bacteria, however, possess defective acid-sensing pathways, making their growth significantly suppressed under low pH conditions. This defective acid-sensing mechanism in fibrolytic bacteria is a core factor contributing to the reduced abundance of fiber-degrading taxa under heat stress (Guan and Liu, 2020).

Meanwhile, heat stress breaks the strictly anaerobic environment of the rumen, leading to elevated ROS levels (Huang et al., 2026). Rumen bacteria sense redox imbalance via oxidation-sensitive transcription factors OxyR, SoxR and PerR, and upregulate antioxidant enzyme genes to counteract oxidative damage. Strictly anaerobic fibrolytic bacteria have an incomplete antioxidant system and are highly sensitive to heat-induced oxidative stress, while facultative anaerobes can survive via activating antioxidant pathways (Choudhary et al., 2024).

HS-induced reduction in dry matter intake alters the amount and composition of fermentable substrates in the rumen. This change is sensed by the cAMP- cAMP receptor protein (CRP) carbon catabolite repression system in rumen bacteria (Görke and Stülke, 2008). Reduced fibrous substrate availability downregulates fibrolytic carbohydrate-active enzyme genes in fibrolytic bacteria and simultaneously upregulates starch-degrading related genes in amylolytic bacteria (Liu et al., 2020). These transcriptional adjustments directly reshape the microbial fermentation profile, and further drive the succession of community structure at the population leve (Wardman et al., 2022).

The core molecular events, functional outcomes and supporting evidence strength of the three sensing pathways are summarized in Table 2. After sensing heat stress signals through the above pathways, rumen microbes initiate adaptive adjustments at the molecular level, which further drive phenotypic changes in community structure and fermentation function.

**Table 2:** Heat stress sensing mechanisms in rumen microbiota.

Sensing pathway category	Specific sensing mechanism	Core mechanism
Direct thermal signal sensing	RNA thermometers	Temperature rise induces RNA conformational change, triggers rapid rpoH translation to activate cellular thermal defense (Kortmann and Narberhaus, 2012; Loh et al., 2018)
Direct thermal signal sensing	σ^32^-mediated heat shock response	Unfolded proteins activate σ^32^ pathway; fibrolytic bacteria with fewer rpoH copies show weaker heat tolerance (Straus et al., 1987; Miwa and Taguchi, 2023)
Direct thermal signal sensing	Two-component system	Membrane kinase senses fluidity changes, transduces thermal signals via phosphorylation to regulate transcription (Jacob-Dubuisson et al., 2018)
Indirect neuroendocrine regulation	Direct cortisol action	Cortisol suppresses rpoH in fibrolytic bacteria, promotes heat-tolerant taxa proliferation and drives community shift (Roncarati and Scarlato, 2017; Syed et al., 2020; Nunez et al., 2025)
Indirect neuroendocrine regulation	Quorum sensing interference	Cortisol disturbs microbial autoinducer secretion and disrupts syntrophic metabolic cooperation (Syed et al., 2020)
Indirect neuroendocrine regulation	Microenvironment remodeling	Splanchnic vasoconstriction impairs rumen anaerobic niche and mucosal defense, inducing microbial dysbiosis (Huang et al., 2018; Meade and O’Farrelly, 2019)
Synergistic microenvironmental sensing	Acid stress	Reduced salivary buffering lowers rumen pH; fibrolytic bacteria with defective acid sensing show growth inhibition (Guan and Liu, 2020; Kim et al., 2022)
Synergistic microenvironmental sensing	Redox imbalance	Elevated ruminal ROS; strictly anaerobic fibrolytic bacteria have incomplete antioxidant systems and weaker stress resistance (Choudhary et al., 2024)
Synergistic microenvironmental sensing	Substrate fluctuation	Altered substrate availability drives CAZyme gene regulation via cAMP-CRP system to reshape fermentation profiles (Liu et al., 2020; Wardman et al., 2022)

### 3.2 Shifts in rumen microbial community composition and functional profiles

Driven by the above molecular sensing and regulatory mechanisms, the rumen microbial community undergoes systematic structural remodeling and functional transformation. This reorganization involves two key disruptions: changes in taxonomic composition and altered functional gene expression. Heat stress significantly remodels the structure of both prokaryotic and protozoan microbial communities.

#### 3.2.1 Phylum-level taxonomic shifts

A notable change at the phylum level is the increased relative abundance of Bacteroidetes, mostly Gram-negative taxa. At the same time, the absolute abundance of Firmicutes (mainly Gram-positive bacteria) and the Firmicutes/Bacteroidetes ratio in the rumen decrease sharply (Park et al., 2022). In dairy cows, HS also reduces the ruminal microbial Firmicutes/Bacteroidetes ratio. This is accompanied by distinct abundance patterns across various microbial taxa (da Silva Pereira et al., 2025). Accumulating evidence demonstrates that the decrease in the Firmicutes/Bacteroidetes ratio is not merely a taxonomic change, but also reflects the functional transformation of the rumen microbiota from fiber-dominated fermentation to non-fibrous carbohydrate-dominated fermentation (Li M. et al., 2026). This shift lays a taxonomic foundation for the subsequent changes in rumen fermentation patterns.

In addition, the abundances of Actinobacteria and Verrucomicrobia also show a significant downward trend under heat stress. Both phyla are involved in the degradation of complex polysaccharides in the rumen and are highly sensitive to rumen pH fluctuations and oxidative environments. Their decreased abundance further weakens the fiber degradation potential of the rumen (Šuľák et al., 2012). Studies on buffalo show different phylum-level change patterns. The abundance of Firmicutes increased, and Proteobacteria and Planctomycetes decreased (Yadav et al., 2022). This interspecies difference may be related to variations in breed heat tolerance, dietary structure and heat stress intensity, reflecting diverse adaptive strategies of rumen microbiota across ruminant species.

#### 3.2.2 Genus-level functional taxon changes

At the genus level, heat stress causes a sharp drop in the relative abundance of fiber-degrading genera. These include *Ruminococcus*, *Desulfovibrio*, *Piromyces*, and *Isotricha*—all cellulolytic microbes vital for hemicellulose degradation and volatile fatty acid (VFA) production (Wang Z. et al., 2021). The abundance of fiber-degrading bacteria decreases by 10.71%, which directly reduces the efficiency of lignin degradation. Protozoa and fungi, which synergize with fibrolytic bacteria to decompose crude fiber, also show 60–80% activity decline under heat stress (Li et al., 2025a). The simultaneous inhibition of bacteria, protozoa and fungi collectively weakens the overall fiber digestion capacity of the rumen.

The imbalance of microbial community structure further forms a local positive feedback effect to aggravate dysbiosis. Studies on Hanwoo cattle show that fibrolytic *Ruminococcaceae* decrease in abundance, while lactogenic *Lactobacillaceae* become more dominant. This microbial imbalance drives a sharp drop in ruminal pH through lactate accumulation. Lactate suppresses fibrolytic activity mainly by disrupting bacterial cell membrane proton gradients and competing for labile carbon substrates. This effect significantly reduces cellulose digestibility and impairs energy harvest efficiency in heat-stressed ruminants (Baek et al., 2020).

On the contrary, heat-tolerant taxa thrive under heat stress. These include *Anaeroplasma* (anaerobic coccoid bacteria for lipid metabolism), *Shuttleworthia* (putative hydrogen-scavenging archaea), and *Filobasidium* (lichenized basidiomycetes with antioxidant properties). Their significant proliferation under heat stress may reflect adaptive responses to oxidative stress and energy substrate reallocation (Park et al., 2022). Chronic heat stress leads to a significant increase in the abundance of *Prevotella*, *Prevotellaceae* and *Planococcaceae* (Li et al., 2024b).

#### 3.2.3 Remodeling of microbial functional potential

At the functional gene level, heat stress downregulates the expression of fibrolytic carbohydrate-active enzyme genes, and simultaneously upregulates genes related to starch and non-fibrous carbohydrate degradation (Onan-Martinez et al., 2026). These changes correspond to the shift in fermentation substrate preference of microbial communities, and provide a functional basis for the subsequent alteration of fermentation patterns.

In addition to carbohydrate-active enzymes, heat stress also leads to extensive reprogramming of rumen microbial energy metabolism and nitrogen metabolism pathways (Raniel et al., 2024). Metagenomic analysis shows that the overall gene expression of core energy metabolic pathways such as glycolysis and tricarboxylic acid cycle is downregulated, while genes related to metabolic pathways of easily available carbohydrates such as starch and sucrose are significantly upregulated, which is fully consistent with the shift of microbial substrate utilization preference (Newbold and Ramos-Morales, 2020). In terms of nitrogen metabolism, the expression levels of microbial urease genes and amino acid synthesis-related genes are significantly reduced, while the expression of protein degradation pathway genes is upregulated. This directly leads to the decline of rumen microbial nitrogen utilization efficiency, which echoes the phenotype of rumen ammonia nitrogen accumulation (Xiong et al., 2024).

Relevant studies have reported that functional genes related to oxidative stress tolerance, including those encoding peroxidase and superoxide dismutase, are significantly upregulated in heat-tolerant microbiota (Wang Z. et al., 2026). The differential expression of these genes is an important molecular basis for the differentiation of heat adaptability among different microbiota, and also confirms the regulatory role of microbial redox sensing pathways. It should be noted that most current studies on rumen microbial function are based on bioinformatics predictions of metagenomics or metatranscriptomics, lacking *in vitro* pure culture verification and direct determination of enzyme activity in the rumen. The specific regulatory mechanisms of related functional changes still need to be further explained.

The key taxonomic shifts of rumen microbiota at different levels and their corresponding physiological consequences under heat stress are comprehensively collated in Table 3. The structural and functional remodeling of rumen microbiota is not only an adaptive response to heat stress, but also the initiating factor of subsequent rumen fermentation dysfunction and epithelial barrier damage, which lays a foundation for the cascade of local lesions to systemic disorders.

**Table 3:** Core changes in rumen microbial community under heat stress in ruminants.

Taxonomic level	Trend under heat stress	Representative genera	Key physiological consequences
Firmicutes	Significantly decreased	Ruminococcus, Fibrobacter	Reduced fiber degradation, decreased acetate production (Guan and Liu, 2020)
Bacteroidetes	Significantly increased	Prevotella	Enhanced starch utilization, elevated propionate proportion (Duan et al., 2025)
Lactate-producing bacteria	Increased	Streptococcus	Lower ruminal pH, higher risk of subacute ruminal acidosis (Hou et al., 2025)
Protozoa & anaerobic fungi	Significantly decreased	Ciliates, anaerobic fungi	Impaired fiber digestion efficiency (reduced by 60–80%) (Baek et al., 2020)
Proteobacteria	Increased	Gram-negative bacteria	Elevated LPS translocation risk (Chen et al., 2021)

### 3.3 Heat stress-induced rumen fermentation dysfunction, barrier disruption and systemic metabolic dysregulation

On the basis of microbial community remodeling, heat stress further disrupts rumen fermentation function and epithelial barrier integrity. Local rumen lesions can be translocated to the whole body through metabolic pathways, immune pathways and neuroendocrine pathways, and form a self-reinforcing vicious cycle with systemic disorders (Jin et al., 2026). As a major global environmental stressor, it severely impairs ruminant production performance, metabolic health and welfare. Its core pathophysiology forms a self-reinforcing vicious cycle that starts with rumen microbial and fermentation disorders, progresses to rumen epithelial barrier damage, and ultimately leads to systemic metabolic and immune failure. Systemic disorders in turn worsen rumen damage and form a reverse exacerbation loop that perpetuates the pathological cycle (Nasrollahi et al., 2019).

#### 3.3.1 Disruption of rumen internal environment homeostasis

Heat stress directly disrupts ruminal fermentation function via two core pathways. One is the disruption of the ruminal microenvironment caused by visceral blood flow redistribution. The other is the disturbance of fermentation substrate supply due to reduced dry matter intake (Uyeno, 2021). Heat stress triggers splanchnic vasoconstriction which reduces rumen epithelial blood flow by 30–40%. This hypoperfusion disturbs the ruminal microenvironment through two main pathways. First, impaired trans-epithelial oxygen exchange breaks the strict anaerobic niche required by beneficial fibrolytic bacteria, and raises reactive oxygen species levels and intraruminal redox potential (Huang et al., 2018). Second, heat stress suppresses the expression of mucins and *β*-defensins in rumen epithelial cells, which weakens innate antimicrobial defense. This leads to overgrowth of Gram-negative bacteria and progressive microbial dysbiosis (Meade and O’Farrelly, 2019).

Elevated ambient temperatures also impair HPA axis function. They suppress salivary gland secretion, reduce saliva output by 40% to deplete bicarbonate buffering capacity, and lower saliva pH from the normal range of 5.8–6.8 to below 5.5 under heat stress (Kinlein et al., 2015). Amylolytic bacteria such as *Streptococcus* spp. overgrow and accelerate starch fermentation in concentrate feeds. This generates excessive lactate (Russell and Hino, 1985). Below pH 5.5, acid-tolerant lactate-utilizing bacteria become inactivated. This prevents the conversion of lactate to propionate, leading to lactic acid accumulation and ultimately resulting in subacute or acute ruminal acidosis (Hou et al., 2025).

Reduced dry matter intake further reshapes rumen fermentation by diminishing fibrous substrates. This change drives the shift of microbial metabolic substrate preference from fiber to non-fibrous carbohydrates. Notably, findings on ruminal pH, total volatile fatty acid and butyrate levels are heterogeneous across published studies. This phenomenon might be caused by the dynamic balance between heat stress duration, microbial volatile fatty acid generation and host epithelial absorption, as well as differences in dietary composition, species sensitivity and experimental conditions (Xu et al., 2013; Naderi et al., 2016).

In addition to local ruminal disturbances, heat stress activates the sympathetic-adrenal medullary system and induces systemic acid–base imbalance. It raises respiratory frequency above 80 breaths per minute and induces compensatory hypocapnia via excessive CO₂ excretion. This worsens systemic alkalosis with blood pH above 7.45 and further impairs hemoglobin buffering capacity (Nakamura et al., 2022), which indirectly aggravates ruminal metabolic disorder by affecting systemic acid–base regulation.

#### 3.3.2 Shifts in rumen fermentation patterns

Fermentation pattern shifts are mainly reflected in changes in volatile fatty acid production and composition. Total volatile fatty acid production decreases by 10 to 30% under heat stress. The proportion of acetate drops from 60 to 70% to below 50%, while propionate becomes the dominant VFA. This marks a shift from acetate-predominant to propionate-predominant fermentation (Bedford et al., 2020).

The VFA metabolic axis serves as a primary pathway for rumen-systemic crosstalk. VFA provide over 70% of total energy requirements for ruminants, and also act as key signaling molecules through free fatty acid receptor 41 (GPR41) and free fatty acid receptor 43 (GPR43), which are widely expressed in host tissues (Naing et al., 2025). HS impairs VFA absorption, which reduces the availability of critical metabolic precursors. These include propionate for hepatic gluconeogenesis, acetate for mammary milk fat synthesis, and butyrate for rumen epithelial energy supply (Wang et al., 2025). Impaired absorption also lowers precursor supply for mammary casein synthesis and reduces milk fat yield (Mu et al., 2021). Delayed VFA clearance elevates plasma non-esterified fatty acid (NEFA) concentrations, which triggers lipid peroxidation and aggravates the vicious cycle of oxidative stress and energy deficit (Adewuyi et al., 2005). The combined effects ultimately drive negative energy balance and reduced production performance in ruminants. Disrupted VFA composition and concentration inhibit insulin secretion from pancreatic *β*-cells and peripheral insulin signaling, inducing systemic insulin resistance that serves as the main driver of widespread glucose and lipid metabolism disorders (Saltiel and Kahn, 2001).

Dysregulated rumen nitrogen metabolism also induces systemic metabolic disturbances via direct circulatory transmission. Under heat stress, microbial nitrogen utilization efficiency decreases, while protein degradation increases. This leads to significant accumulation of ruminal ammonia nitrogen (Shah et al., 2020). Excess free ammonia gains entry into the systemic circulation, yet heat stress compromises hepatic urea cycle activity, precluding the efficient conversion of ammonia to urea and thereby leading to hyperammonemia. Elevated blood ammonia concentrations impair both hepatic and renal function, disrupt central nervous system control over feeding behavior and energy metabolism, and exacerbate preexisting systemic metabolic perturbations (Raniel et al., 2024).

#### 3.3.3 Ruminal and gut barrier dysfunction and endotoxin translocation

Gastrointestinal epithelial barrier breakdown and subsequent endotoxin translocation represent a core pathological pathway that connects local ruminal fermentation dysfunction to systemic health impairment in ruminants.

Under HS, barrier damage does not occur exclusively in the rumen. It spreads across the entire digestive tract and leads to a pathological state known as leaky gut. This state features elevated intestinal permeability and the loss of the epithelium’s selective barrier function (Mishra et al., 2025). In this state, luminal toxins, bacterial antigens and microbial metabolites can translocate across the mucosa into the lamina propria and systemic circulation, triggering systemic inflammatory and stress responses (Wang M. Y. et al., 2021; Fang et al., 2023). The gastrointestinal microbiota operates as a continuous, interconnected ecosystem instead of functioning as separate, independent units. Changes in ruminal microbial composition alter the substrate supply in downstream digesta, which in turn reshapes the structure and function of microbiota in the lower gut and feces. In reverse, colonic microbial dysbiosis amplifies systemic pathological damage through the leaky gut phenotype.

Fermentation dysfunction compromises epithelial integrity through several synergistic mechanisms. These include drastic pH fluctuations, insufficient butyrate supply that cuts off the core energy source for epithelial cells, oxidative damage caused by reactive oxygen species, and pre-existing ruminal microbial dysbiosis (Son et al., 2023). These ruminal microbial alterations further drive systemic metabolic disorders, activate bacterial LPS synthesis pathways, and downregulate tight junction protein expression in the small intestine and colon, extending barrier impairment to the entire lower digestive tract (Li Z. et al., 2026). Mounting evidence supports that dysbiosis of either the ruminal or intestinal microbiota impairs mucosal barrier integrity. This impairment allows bacteria and their metabolic products to translocate to extraintestinal organs at distal sites (Hu et al., 2024).

When the epithelial barrier is compromised, luminal LPS released by Gram-negative bacteria enters the portal circulation. It then activates the TLR4/NF-κB signaling pathway in hepatic Kupffer cells, which prompts a large release of pro-inflammatory cytokines such as TNF-*α*, IL-1β and IL-6 (Liu et al., 2026). This signaling cascade leads to metabolic endotoxemia and chronic low-grade systemic inflammation. These conditions in turn worsen insulin resistance, systemic oxidative stress, and metabolic dysfunction across multiple organs (Chen et al., 2021). Endotoxin exposure also drives oxidative stress by stimulating reactive oxygen species production in inflammatory cells and other cell types, creating a mutually reinforcing cycle between inflammation and oxidative damage (Chirivi and Contreras, 2024). The widespread leaky gut phenotype along the entire gastrointestinal tract has effects beyond driving systemic inflammation. It further disrupts whole-body metabolic homeostasis, including aggravated peripheral insulin resistance, impaired hepatic nutrient metabolism, and reduced milk synthesis efficiency in dairy cows (Ellett et al., 2024).

Notably, cortisol and catecholamines released by the activated neuro-endocrine stress axis can worsen gastrointestinal epithelial barrier dysfunction and microbial dysbiosis in return. This forms a systemic self-amplifying vicious cycle that sustains the pathological damage induced by heat stress.

#### 3.3.4 Self-reinforcing vicious cycle between rumen dysfunction and systemic disorders

The neuro-endocrine-immune network acts as a crucial mediator for long-distance bidirectional crosstalk between the rumen and systemic physiological processes. Rumen-derived LPS, inflammatory signals, and metabolic disturbances collectively trigger activation of the HPA axis and sympathetic nervous system. Cortisol, the key stress hormone, accumulates persistently in the circulation. This promotes mobilization of body protein and fat, exacerbating negative energy balance (Agwaan, 2026). It also enhances splanchnic vasoconstriction, reducing rumen epithelial blood flow and amplifying initial rumen damage through a feed-forward loop. In turn, systemic metabolic, inflammatory, and oxidative disorders worsen rumen dysfunction, forming a self-reinforcing vicious cycle (Zhang et al., 2022).

Systemic negative energy balance redirects blood flow toward vital organs, which in turn exacerbates rumen ischemia by reducing blood supply to the rumen (Wang et al., 2026a). Circulating pro-inflammatory cytokines exert a direct downregulatory effect on tight junction proteins in the rumen epithelium, while systemic reactive oxygen species disrupt the rumen’s strict anaerobic milieu, perpetuating microbial dysbiosis and impairing fermentation processes (Wang Z. et al., 2021).

In summary, rumen-systemic crosstalk under heat stress is driven by interconnected mechanistic pathways. The self-amplifying cycle of “local rumen microbial fermentation disorder → epithelial barrier damage → systemic metabolic inflammation → further rumen function deterioration” is the core pathological feature of heat stress injury in ruminants. Elucidating these mechanisms provides critical theoretical targets for nutritional intervention to mitigate heat stress damage in ruminants.

## 4 Rumen-host crosstalk: An amplifier of systemic pathology in heat-stressed ruminants

**Figure 3:**
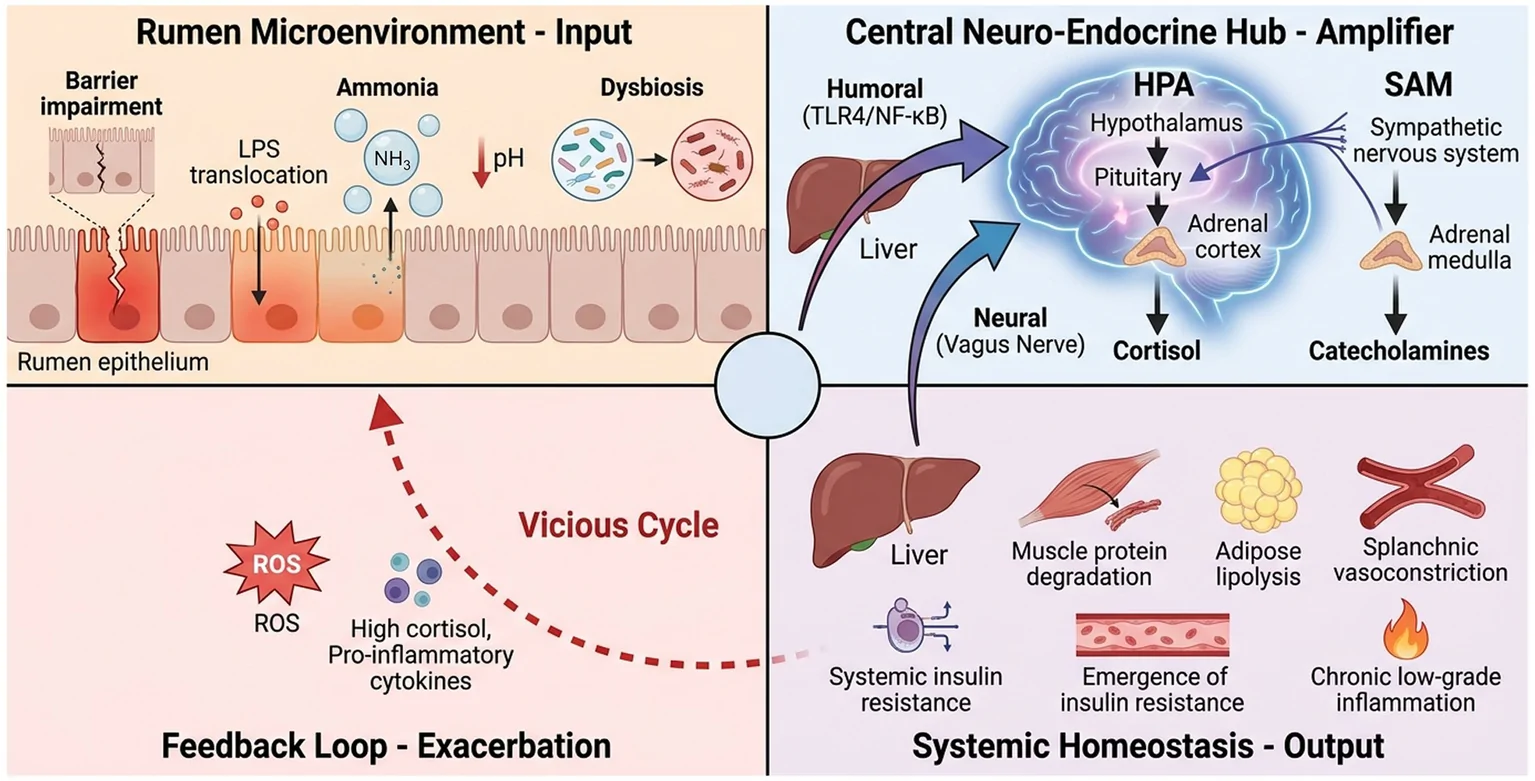
Heat stress-induced rumen dysbiosis: The mediating role of neuro-endocrine-immune crosstalk. All schematics were designed by the authors based on evidence summarized in this review and drawn using BioGDP (https://www.biogdp.com/) and Microsoft PowerPoint (Microsoft Corp., Redmond, WA, USA). No new experimental data were generated for this review. Comprehensive mechanistic descriptions and supporting references are provided in the main body of the manuscript.

The neuro-endocrine-immune network dominates bidirectional crosstalk between rumen microenvironment and systemic physiological homeostasis and acts as a pathological amplifier forming a self-reinforcing vicious cycle, as depicted in Figure 3. The schematic depicts the delivery of rumen-derived LPS, inflammatory cytokines and metabolic disruptions to central HPA and SAM axes through humoral and vagal afferent signaling. Circulating cortisol and catecholamines induced by central stress activation drive systemic metabolic and inflammatory anomalies, while concurrently generating a negative feedback loop that aggravates ruminal ischemia, microbial imbalance and epithelial barrier damage. This self-reinforcing circuit visually substantiates the neuro-endocrine-immune network as an active pathological amplifier capable of translating localized ruminal damage into persistent systemic pathological deterioration.

Accumulating evidence supports that the neuro-endocrine-immune network dominates bidirectional crosstalk between rumen microenvironment and systemic physiological homeostasis, serving as the core hub and amplifier that drives localized ruminal fermentation dysfunction into multi-systemic metabolic collapse. This regulatory cascade adheres to a progressive pathological logic. It begins with the initiation of ruminal pathological signals, followed by amplification through central stress axes, then systematic disruption of bodily homeostasis, and ultimately leads to reciprocal exacerbation of the initial ruminal damage (Bradford et al., 2016). Such self-sustaining circulation enables long-distance transmission of local gastrointestinal lesions and facilitates irreversible pathological deterioration, which fundamentally accounts for compromised productive performance and elevated incidence of metabolic disorders in heat-stressed ruminants.

### 4.1 Afferent pathways: dual transmission of rumen-derived pathological signals

The afferent delivery of ruminal pathological signals to the central nervous system functions as the initial trigger of the entire stress cascade (Yu et al., 2020). Under heat stress, distinctive detrimental alterations within the rumen are transmitted to central regulatory centers through two coordinated and complementary pathways, with varying levels of supporting evidence for each route.

The humoral hepato-cerebral pathway allows translocated LPS, excessive ammonia nitrogen and inflammatory mediators to enter the liver via portal circulation after ruminal barrier impairment (Sarmikasoglou et al., 2024). These hazardous substances activate the hepatic TLR4/NF-κB inflammatory cascade and induce local oxidative damage, triggering sequential secretion of TNF-*α*, IL-1β and IL-6. These cytokines enter systemic circulation and regulate hypothalamic function through two routes: they induce cerebrovascular endothelial cells to produce secondary inflammatory signals that cross the blood–brain barrier, or rapidly activate central microglia via vagal afferents to mediate neuroinflammation and HPA axis sensitization (Fan et al., 2023; Ohara and Hsiao, 2025).

The vagus nerve provides a rapid neural transmission route, in which abundant afferent sensory fibers distributed in ruminal mucosa express pattern recognition receptors and metabolite receptors including TLR4 and GPR43 directly perceive intraruminal pH fluctuation, endotoxin accumulation and redox imbalance (Xing et al., 2026). Perceptual signals are immediately delivered to the hypothalamic paraventricular nucleus through the nucleus tractus solitarii, establishing a rapid neurogenic connection between ruminal microenvironmental disorder and central stress response (Li et al., 2024a). Disordered rumen microbial metabolites also participate in afferent signal transmission. Reduced short-chain fatty acids weaken barrier protection and neuromodulation via corresponding receptors, while tryptophan metabolite imbalance interferes with mucosal immunity and neural signaling. Altered secondary bile acids further worsen hepatic and central metabolic dysregulation. Together, these bioactive molecules form the molecular foundation of rumen-host crosstalk. The hypothalamus receives pathological signals from the rumen. It then activates central stress-related signaling systems, which amplify adverse stimuli and convert localized gastrointestinal lesions into systemic stress responses (Oh et al., 2013).

Multi-omics studies offer correlative systematic support for this regulatory network. Integrated metagenomic and metabolomic analyses identify key microbial strains and metabolic markers mediating rumen-systemic interactions, and hypothalamic transcriptomics maps the full transcriptional landscape of central signal integration.

### 4.2 Central integration: neuro-endocrine-immune mediation of systemic dysregulation

The hyperactivated HPA axis elevates circulating cortisol, compromising rumen epithelial barrier function (Matsuzaki et al., 2023). The sympathetic-adrenomedullary system is concurrently activated to facilitate massive catecholamine secretion from adrenal medulla (Carter and Goldstein, 2015). The two central stress axes act in synergy. Cortisol mediates long-lasting metabolic and immunomodulatory adjustments, while catecholamines dominate rapid cardiovascular and visceral circulatory responses. Circulating cortisol and catecholamines are key stress mediators. They serve as a crucial molecular basis for the subsequent physiological dysfunction and metabolic disorders that affect multiple organs and tissues (Liu et al., 2021).

Sustained accumulation of stress-related hormones disrupts the homeostasis of energy metabolism, visceral circulation and immune defense through extensive molecular regulation, ultimately leading to systematic physiological deterioration (Wang et al., 2024). Cortisol regulates peripheral metabolic patterns by facilitating skeletal muscle protein degradation and adipose lipolysis through FoxO1-mediated proteolytic and ATGL-driven lipolytic pathways. It aggravates negative energy balance, impairs peripheral insulin sensitivity by blunting PI3K/Akt insulin signaling cascades, and thereby induces systemic insulin resistance and glucose-lipid metabolic disorders (Rigal et al., 2024). Catecholamines bind to adrenergic receptors on vascular smooth muscle, triggering marked splanchnic vasoconstriction. Cortisol reinforces vascular responsiveness to stress hormones, reduces blood flow to the ruminal epithelium, and thus induces sustained ischemia and hypoxia in ruminal tissues. Hyperactive sympathetic tone and excessive glucocorticoid secretion disrupt immune homeostasis by overactivating innate inflammatory responses and suppressing adaptive immune function, consequently causing chronic low-grade inflammation and weakened disease resistance in heat-exposed ruminants (Wang et al., 2022).

The intensity of stress response and systemic dysregulation varies across species and physiological stages. Studies have shown that peak-lactation high-yield dairy cows show the lowest HPA activation threshold and most severe metabolic disturbance, with elevated risk of concurrent ketosis and ruminal acidosis (Stefanska et al., 2024). Goats exhibit stronger heat tolerance than sheep, with milder barrier damage and inflammatory response under identical heat exposure (Aboulnaga et al., 2025). This systemic pathological cascade directly translates to productive losses. Systemic insulin resistance and inhibited mammary protein synthesis reduce milk protein output, while disordered lipid metabolism lowers milk fat content. Chronic low-grade inflammation also increases the incidence of periparturient metabolic diseases, causing substantial economic losses to production systems.

### 4.3 Vicious cycle: systemic feedback exacerbates rumen lesions

Systemic deterioration in metabolism, inflammation and redox balance is not merely a passive consequence of heat stress, but reciprocally exacerbates ruminal lesions and consolidates a self-reinforcing vicious cycle (Che et al., 2026). Severe negative energy balance leads to adaptive blood redistribution, which prioritizes the blood supply of vital organs, aggravates visceral ischemia, and inhibits the proliferation and repair ability of rumen epithelial cells.

Pro-inflammatory mediators in the circulation impair the integrity of the rumen barrier, downregulate the expression of tight junction proteins via myosin light chain kinase-dependent pathways, and induce epithelial cell apoptosis through mitochondrial and endoplasmic reticulum stress pathways (Thanga Velu et al., 2026). Meanwhile, these pro-inflammatory mediators reduce the transport efficiency of ruminal volatile fatty acids by downregulating epithelial monocarboxylate transporters and exacerbate fermentation disorders (Akhtar et al., 2022). Excessive systemic reactive oxygen species diffuse into the rumen and elevate intraruminal redox potential, inhibiting the proliferation of obligate anaerobic fibrolytic bacteria and shifting microbial community structure from fibrolytic to amylolytic dominance. This disturbance in microbial community composition further exacerbates ruminal microbial dysbiosis (Li et al., 2025b). Circulating cortisol directly binds to glucocorticoid receptors in ruminal epithelium to weaken mucosal defense and antimicrobial capacity by suppressing antimicrobial peptide secretion, thereby facilitating the overgrowth of Gram-negative bacteria and continuous endotoxin accumulation within the rumen (Aschenbach et al., 2019). This reciprocal exacerbation demonstrates that the rumen is not merely a passive victim of heat stress, but rather an active amplifier of systemic pathology through the neuro-endocrine-immune network.

The key biological events and corresponding host outcomes at each stage of the self-reinforcing rumen-systemic vicious cycle are clearly sorted out in Table 4. Targeted nutritional interventions that interrupt this self-reinforcing cycle at three key nodes provide indirect functional support for the amplifier model. Stabilizing the ruminal environment and protecting epithelial barrier function reduces pathological signal generation. Regulating stress axis excitability with functional amino acids or plant extracts attenuates central amplification of adverse stimuli. Supplementing antioxidants and anti-inflammatory substances suppresses systemic disorder and promotes rumen epithelial repair.

**Table 4:** Key nodes of the rumen-systemic vicious cycle induced by heat stress.

Cycle Stage	Key Biological Events	Host Outcomes
Initiation	Heat stress → reduced DMI and saliva secretion	Declined buffering capacity, low ruminal pH (Son et al., 2023)
Rumen injury	Microbial dysbiosis, epithelial barrier disruption	LPS entry into blood, abnormal VFA absorption (Bedford et al., 2020)
Central activation	Humoral and vagal signals stimulate HPA/SAM axis	Sustained high cortisol and catecholamines (Gonzalez-Rivas et al., 2020)
Systemic disorder	Oxidative stress, inflammation, insulin resistance	Lower productivity, metabolic disorders (Rigal et al., 2024)
Vicious cycle	Systemic stress aggravates rumen ischemia and dysbiosis	Progressive pathological amplification (Wang Z. et al., 2021)

## 5 Mitigation strategies targeting rumen-host crosstalk under heat stress

The self-amplifying vicious cycle between rumen dysfunction and systemic pathology forms the core mechanism of heat stress damage in ruminants. Interventions targeting key nodes of this cycle can effectively block signal initiation, cascade amplification and reverse exacerbation, thereby mitigating production losses and health impairment. Currently, the main intervention measures include targeted nutritional regulation, feeding management optimization and genetic improvement. Among them, nutritional strategies are the most widely studied and feasible approaches in production practice, with action targets covering the whole cycle of rumen-host crosstalk.

### 5.1 Ruminal-targeted nutritional interventions: blocking the initiation of pathological signals

Stabilizing the ruminal microenvironment, restoring microbial homeostasis and protecting epithelial barrier integrity can cut off the source of pathological signals such as LPS and abnormal metabolites, which is the primary strategy to interrupt the amplifier cycle from the initiation end.

Probiotics and direct-fed microbials are the most commonly used rumen-targeted regulators, which optimize community structure by supplementing beneficial bacteria, and enhance barrier function by promoting epithelial cell repair. Lactic acid bacteria enriched in fermented feed can improve the rumen fermentation pattern, increase the proportion of volatile fatty acids supplied to mammary energy metabolism, and enhance nutrient partitioning efficiency in heat-stressed ruminants (Zhang et al., 2020). This *in vivo* ruminant study provides strong direct evidence for rumen-targeted intervention. Several other strains have also been validated directly in ruminant models. *Clostridium butyricum* effectively alleviates the adverse effects of heat stress in goats, by improving rumen fermentation and growth performance (Xue et al., 2022). *Saccharomyces cerevisiae* improves feed efficiency in dairy cows (Kumar et al., 2023). One study reported that dairy cows fed 50 grams of *Saccharomyces cerevisiae* per day had rumen temperatures roughly 0.2 °C lower than the control group (Lees et al., 2021).

In contrast, some strains show promising effects in monogastric animals, but lack direct in vivo verification in ruminants. *Lactobacillus plantarum L19*, isolated from Holstein cow milk, has been verified to reduce HSP70 levels, pro-inflammatory cytokine release and hepatic oxidative stress in heat-stressed mice by modulating intestinal microbiota (Wang et al., 2026b). Similarly, *Lactiplantibacillus plantarum* supplementation alleviates heat stress-induced testicular injury via regulation of the microbiota-gonad axis (Tu et al., 2026). These observations demonstrate the general stress-alleviating capacity of lactic acid bacteria. Nevertheless, in vivo trials exploring their regulatory roles in the ruminal microecology, epithelial barrier integrity and lactation performance of heat-stressed dairy cows are scarce. Large-scale feeding trials are therefore required to validate their practical application potential in ruminant husbandry.

*Bacillus species* have been shown to alleviate oxidative and physiological responses to heat stress in hens by reshaping gut microbiota structure (Suzuki et al., 2026). As spore-forming probiotics, Bacillus strains have strong tolerance to the harsh ruminal environment. This makes them well-suited for regulating rumen microecology in heat-stressed ruminants, with good application prospects. Direct *in vivo* data from dairy cows support this potential. For lactating dairy cows, milk production increased linearly as dietary *Bacillus subtilis* supplementation levels rose gradually (Choonkham et al., 2020).

In general, rumen-targeted interventions act on the first node of the amplifier cycle, reducing the generation and translocation of rumen-derived pathological signals by improving microbial homeostasis and barrier function.

### 5.2 Systemic regulatory nutritional interventions: suppressing cascade amplification and reverse exacerbation

Systemic oxidative stress and chronic low-grade inflammation are not only core manifestations of heat stress-induced systemic disorders, but also key mediators that reversely aggravate rumen damage (Yang Z. et al., 2025). Antioxidant and anti-inflammatory nutritional strategies can suppress the systemic inflammatory cascade on the one hand, and reduce oxidative damage to rumen epithelium and disruption of the anaerobic microenvironment on the other hand, thus blocking both the amplification and reverse exacerbation links of the cycle.

Plant-derived bioactive compounds are widely explored for heat stress mitigation due to their high bioactivity, low toxicity and multi-target regulatory properties. As a typical polyphenolic active substance, resveratrol has shown protective effects in both ruminant production trials and cellular mechanistic studies (Wei et al., 2026). Dietary resveratrol supplementation increases plasma total antioxidant capacity, alleviates systemic oxidative damage and mitigates production loss in heat-stressed dairy cows, making it a promising feed additive for commercial dairy farms under thermal stress (Nair et al., 2026). Trials on early-lactation Holstein cows also confirm that resveratrol supplementation exerts positive effects on feed intake and lactation performance under heat stress (Ahvanooei et al., 2025).

At the molecular level, resveratrol regulates cellular stress defense and metabolic homeostasis mainly via activating the SIRT1/AMPK signaling pathway, and protects reproductive cells by reducing oxidative damage, preserving mitochondrial structural integrity and inhibiting cell apoptosis (Tariq et al., 2025). In addition, resveratrol possesses broad antioxidant, anti-inflammatory, anti-obesity and antimicrobial activities that contribute to improved reproductive performance. Its modulation of rumen microbial communities can also significantly reduce rumen methane emissions, bringing additional environmental benefits to ruminant production (Rezaei Ahvanooei et al., 2023).

Melatonin, another bioactive molecule with strong free radical scavenging capacity, also shows application potential in alleviating heat stress damage (Gharanjik et al., 2025). Goat model studies have verified that melatonin reverses heat stress-induced tricarboxylic acid cycle suppression and mitochondrial damage in Sertoli cells, thus maintaining homeostasis of the spermatogenic microenvironment (Yang G. et al., 2025). Naringenin exerts antioxidant and anti-inflammatory effects by modulating the Nrf2/Keap1 pathway, enhancing the body’s total antioxidant capacity, inhibiting the excessive release of pro-inflammatory factors, and thus attenuating heat stress-induced thymic injury in chickens (Liao et al., 2026). Essential oil blend supplementation can mitigate heat stress and improve growth performance in growing crossbred lambs by enhancing systemic antioxidant capacity (Ma et al., 2026). This *in vivo* study in lambs provides moderate direct evidence for the application of plant extracts in ruminants.

In addition to systemic regulation, essential oils can also directly regulate rumen microbiota composition and improve fermentation efficiency, playing a dual regulatory role on both local rumen and systemic levels (Kaewkaen, 2026). Supplementing antioxidant precursors or trace elements related to antioxidant enzyme activity can directly enhance the body’s own antioxidant defense system. N-acetyl cysteine (NAC), as a precursor of glutathione, can effectively improve growth performance, serum biochemical indexes, antioxidant capacity and hepatic morphology in heat-stressed broilers, and regulate the expression of heat shock protein-related genes (El-Barbary et al., 2026). Selenium (Se) is a core component of glutathione peroxidase. Dietary supplementation with an appropriate dose of Se can ameliorate heat stress-induced negative effects on oxidative status, hormonal balance, cytokine profile and immune function in goats, and maintain Se metabolism homeostasis (Aderao et al., 2025). This in vivo ruminant study provides strong evidence for antioxidant intervention to alleviate heat stress damage.

Such systemic regulatory strategies act on the middle and downstream nodes of the amplifier cycle. By alleviating systemic oxidative inflammation and metabolic disorders, they reduce reverse damage to the rumen and weaken the self-amplification effect of the cycle.

### 5.3 Management optimization and genetic breeding strategies: reducing heat stress load from the source

In addition to nutritional interventions, environmental management and genetic improvement are fundamental strategies to cope with heat stress, which can reduce the intensity of heat stress stimulation from the source, thereby weakening the starting power of the amplifier cycle.

Physical cooling measures such as spray cooling, mechanical ventilation and shading shed construction can directly reduce the temperature and humidity of the breeding environment and lower the core body temperature of animals (Correa-Calderon et al., 2004; Levit et al., 2021). A recent study has reported that the geothermal heat pump - precise air supply (GHP-PAS) system can alleviate the heat stress of lactating dairy cows (Li et al., 2025c). Adjusting feeding time to cool periods (morning and evening), increasing feeding frequency, and ensuring sufficient clean drinking water can alleviate the decrease in dry matter intake caused by heat stress, and indirectly maintain the stability of the rumen internal environment (Curtis et al., 2017).

Breeding ruminant breeds with strong heat tolerance through genetic selection is a long-term fundamental solution (Neufeldt et al., 2026). By screening heat-tolerant germplasm resources and mining key genes related to heat tolerance, we can cultivate breeds with both high production performance and heat adaptability (Capela et al., 2026). From the perspective of rumen-host crosstalk, selecting individuals with stable rumen microecology and low inflammatory response under heat stress can effectively reduce the amplification efficiency of the pathological cycle at the genetic level.

Collectively, current mitigation strategies cover multiple key nodes of the rumen-host crosstalk amplifier cycle, among which nutritional regulation has the strongest operability and widest application in production practice. However, most existing studies only focus on the improvement of production performance or single physiological indexes, and lack systematic verification of intervention effects on the whole bidirectional crosstalk cycle. In addition, there is still a lack of optimized combined intervention schemes targeting multiple nodes simultaneously, which is an important direction for future research.

In conclusion, the neuro-endocrine-immune crosstalk acts as a critical molecular switch linking localized ruminal damage to systemic pathological lesions and mediates the progression from acute heat stress to chronic persistent injury. Intricate signal transduction and cascade amplification serve as a key link, connecting ruminal fermentation disturbance, epithelial barrier defect and microbial dysbiosis to systemic energy dysregulation, inflammatory outburst and oxidative imbalance. This interconnection forms a closed, irreversible pathological circulation, which is consistent with the pathological characteristics of ruminant metabolic disorders. This regulatory mechanism fully clarifies the gastrointestinal origin of systematic physiological abnormalities in heat-stressed ruminants. In-depth interpretation of such interactive pathways can provide theoretical references for developing targeted nutritional regulation strategies and screening anti-stress additives, so as to effectively alleviate heat stress-induced production losses and health hazards in ruminant production.

## 6 Conclusion

Heat stress causes progressive, self-amplified pathological damage in ruminants. The bidirectional crosstalk between the host systemic stress response and rumen microbiota, with the neuro-endocrine-immune network as the core hub and pathological amplifier, forms the core pathological basis. Specifically, hyperactivation of the HPA and SAM axes drives systemic oxidative stress, immune dysfunction and chronic low-grade inflammation, and indirectly disrupts rumen homeostasis via neuroendocrine signals and splanchnic vasoconstriction. In turn, rumen microbiota perceives heat stress through three synergistic pathways, and the resulting microbial dysbiosis, fermentation disorder and epithelial barrier damage further aggravate systemic pathological disorders through humoral and vagal afferent pathways, forming a self-sustaining vicious cycle. This mechanism reveals the ruminal origin of systemic heat stress damage in ruminants.

Current research still faces limitations including insufficient causal evidence, unclear contribution of different pathways, and limited cross-species data. Future studies should focus on verifying the causal role of the neuro-endocrine-immune network in rumen-host crosstalk, quantifying the contribution of core pathways, and developing multi-node intervention strategies targeting the amplification cycle. In-depth interpretation of this regulatory mechanism will provide a systematic theoretical basis for alleviating heat stress-related production losses and supporting sustainable ruminant farming.
